# Molecular insights into the interaction between human nicotinamide phosphoribosyltransferase and Toll-like receptor 4

**DOI:** 10.1016/j.jbc.2022.101669

**Published:** 2022-02-02

**Authors:** Massimiliano Gasparrini, Francesca Mazzola, Massimiliano Cuccioloni, Leonardo Sorci, Valentina Audrito, Federica Zamporlini, Carlo Fortunato, Adolfo Amici, Michele Cianci, Silvia Deaglio, Mauro Angeletti, Nadia Raffaelli

**Affiliations:** 1 Department of Agricultural, Food and Environmental Sciences, Polytechnic University of Marche, Ancona, Italy; 2 Department of Clinical Sciences, Polytechnic University of Marche, Ancona, Italy; 3 School of Biosciences and Veterinary Medicine, University of Camerino, Camerino, Italy; 4 Division of Bioinformatics and Biochemistry, Department of Materials, Environmental Sciences and Urban Planning, Polytechnic University of Marche, Ancona, Italy; 5 Department of Medical Sciences, University of Turin, Turin, Italy

**Keywords:** NAD biosynthesis, Toll-like receptor 4 (TLR4), NAMPT, receptor-interacting protein (RIP), surface plasmon resonance (SPR), inflammation, DAMP, CCR5, chemokine receptor type 5, DAMP, damage-associated molecular pattern, LPS, lipopolysaccharide, MD2, myeloid differentiation factor 2, Nam, nicotinamide, NAMPT, nicotinamide phosphoribosyltransferase, NAPRT, nicotinate phosphoribosyltransferase, NMN, nicotinamide mononucleotide, PRPP, phosporibosyl pyrophosphate, SEAP, secreted alkaline phosphatase, SPR, surface plasmon resonance, TLR4, Toll-like 4 receptor

## Abstract

The secreted form of the enzyme nicotinamide phosphoribosyltransferase (NAMPT), which catalyzes a key reaction in intracellular NAD biosynthesis, acts as a damage-associated molecular pattern triggering Toll-like receptor 4 (TLR4)-mediated inflammatory responses. However, the precise mechanism of interaction is unclear. Using an integrated approach combining bioinformatics and functional and structural analyses, we investigated the interaction between NAMPT and TLR4 at the molecular level. Starting from previous evidence that the bacterial ortholog of NAMPT cannot elicit the inflammatory response, despite a high degree of structural conservation, two positively charged areas unique to the human enzyme (the α1-α2 and β1-β2 loops) were identified as likely candidates for TLR4 binding. However, alanine substitution of the positively charged residues within these loops did not affect either the oligomeric state or the catalytic efficiency of the enzyme. The kinetics of the binding of wildtype and mutated NAMPT to biosensor-tethered TLR4 was analyzed. We found that mutations in the α1-α2 loop strongly decreased the association rate, increasing the *K*_D_ value from 18 nM, as determined for the wildtype, to 1.3 μM. In addition, mutations in the β1-β2 loop or its deletion increased the dissociation rate, yielding *K*_D_ values of 0.63 and 0.22 μM, respectively. Mutations also impaired the ability of NAMPT to trigger the NF-κB inflammatory signaling pathway in human cultured macrophages. Finally, the involvement of the two loops in receptor binding was supported by NAMPT-TLR4 docking simulations. This study paves the way for future development of compounds that selectively target eNAMPT/TLR4 signaling in inflammatory disorders.

By catalyzing a key reaction in the NAD biosynthetic pathway starting from nicotinamide, the enzyme nicotinamide phosphoribosyltransferase (NAMPT) represents the key enzyme for the maintenance of steady-state NAD levels. In fact, the nicotinamide moiety released from NAD by all intracellular NAD-consuming enzymes (*i.e.*, sirtuins, ADP ribosyltransferases, and NAD glycohydrolases) is recycled back to the coenzyme by NAMPT that phosphoribosylates the pyridine base to nicotinamide mononucleotide (NMN). In a subsequent reaction, NMN is adenylated to NAD by the enzyme NMN adenylyltransferase. Several cells, including immune cells, cancer cells, and adipocytes, can actively release NAMPT into the extracellular space, with different mechanisms depending on the cell type (1, 2, 3, 4). Once secreted, the extracellular enzyme (eNAMPT) acts as a cytokine-like protein, triggering intracellular signaling pathways that result in a wide range of different effects: increased aggressiveness in cancer cells, enhanced functionality in pancreatic β-cells and endothelial cells, and proinflammatory effects in immune cells (5, 6). Stressful conditions like starvation, hypoxia, oxidative stress, and inflammation promote the enzyme's release, but whether the enzyme's secretion also occurs under physiological conditions is still unclear. Indeed, basal levels of circulating NAMPT can be detected in healthy subjects, and they markedly increase in cancer and inflammatory diseases, confirming a role of the secreted enzyme in these conditions (7, 8). Accordingly, administration of NAMPT-neutralizing antibodies attenuates the inflammatory response in several preclinical models, including bowel disease and lung injury (9, 10).

The molecular mechanism of eNAMPT action has not been clarified yet. Evidence is provided that the protein might exert its cytokine function by binding to a cell membrane receptor. In particular, eNAMPT secreted by macrophages at the site of the skeletal muscle injury is reported to stimulate myoblasts proliferation in a C-C chemokine receptor type 5 (CCR5)-dependent manner (11). Also, eNAMPT binds CCR5 in cancer cells and acts as an antagonist of the receptor (12). Accordingly, a direct interaction of NAMPT with CCR5 has been confirmed through surface plasmon resonance (SPR) and ELISA analyses, by using the human recombinant proteins (11, 13). However, the finding that eNAMPT also activates non-CCR5-dependent pathways, like the NF-κB pathway, suggests that the protein might recognize other types of receptors. Indeed, accumulating evidence points to a significant role of Toll-like 4 receptor (TLR4) in eNAMPT signaling. The Garcia group first showed that the TLR4-dependent activation of the NF-κB pathway involved in lung inflammatory events is mediated by circulating eNAMPT and demonstrated a physical interaction between recombinant NAMPT and the extracellular domain of TLR4 through SPR analysis (14). In subsequent studies, the eNAMPT/TLR4 signaling was found to contribute to endothelial dysfunction and vascular inflammation in murine microvessels (15). Our previous work also established that eNAMPT drives the transcription and secretion of several proinflammatory cytokines in human macrophages by triggering the TLR4-dependent activation of the NF-κB pathway (16). Indeed, in TLR4-silenced macrophages and in macrophages from TLR4^−/−^ mice, exposure to NAMPT was not able to activate the inflammatory response (16). Altogether, these data identify eNAMPT as a novel damage-associated molecular pattern (DAMP) protein, priming immune and nonimmune cells.

In this work, we have dissected the interaction between NAMPT and the extracellular domain of TLR4 at the molecular level by combining bioinformatic, structural, and functional analyses. We identified two regions in human NAMPT involved in TLR4 binding that might be targeted to impair eNAMPT/TLR4 signaling.

## Results

### Identification of NAMPT structural signatures as potential signaling determinants

**Figure 1:**
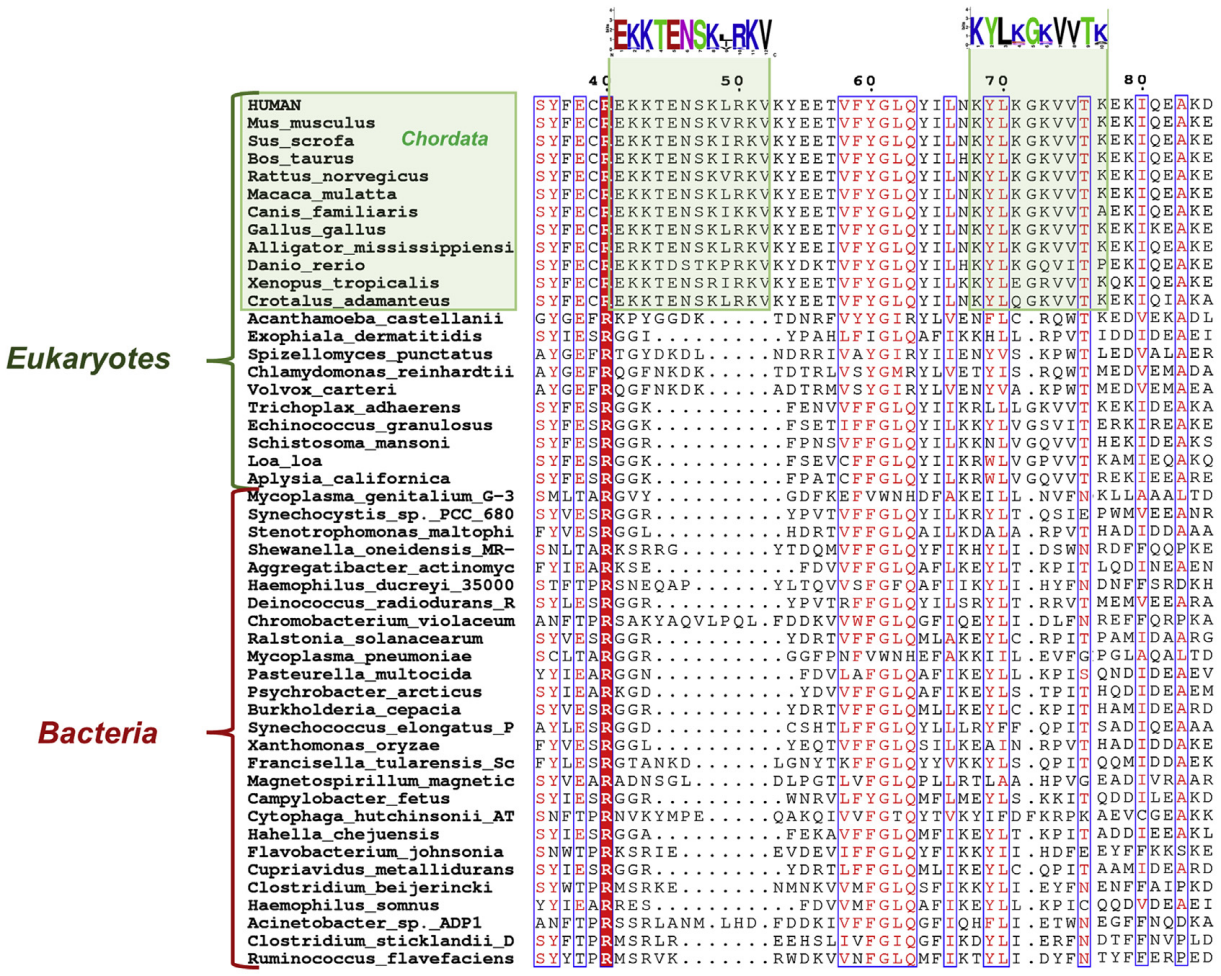
**Section of multiple sequence alignment of NAMPTs from 50 diverse eukaryotes (including lower unicellular organisms) and bacteria.** Sequence numbering is according to human NAMPT. Partially conserved residues (numbered by *Hs*NAMPT sequence) are *boxed*, and universally conserved residues are highlighted by a *solid background red color*. Possible regions implicated in signaling are shaded in *green*.

**Figure 2:**
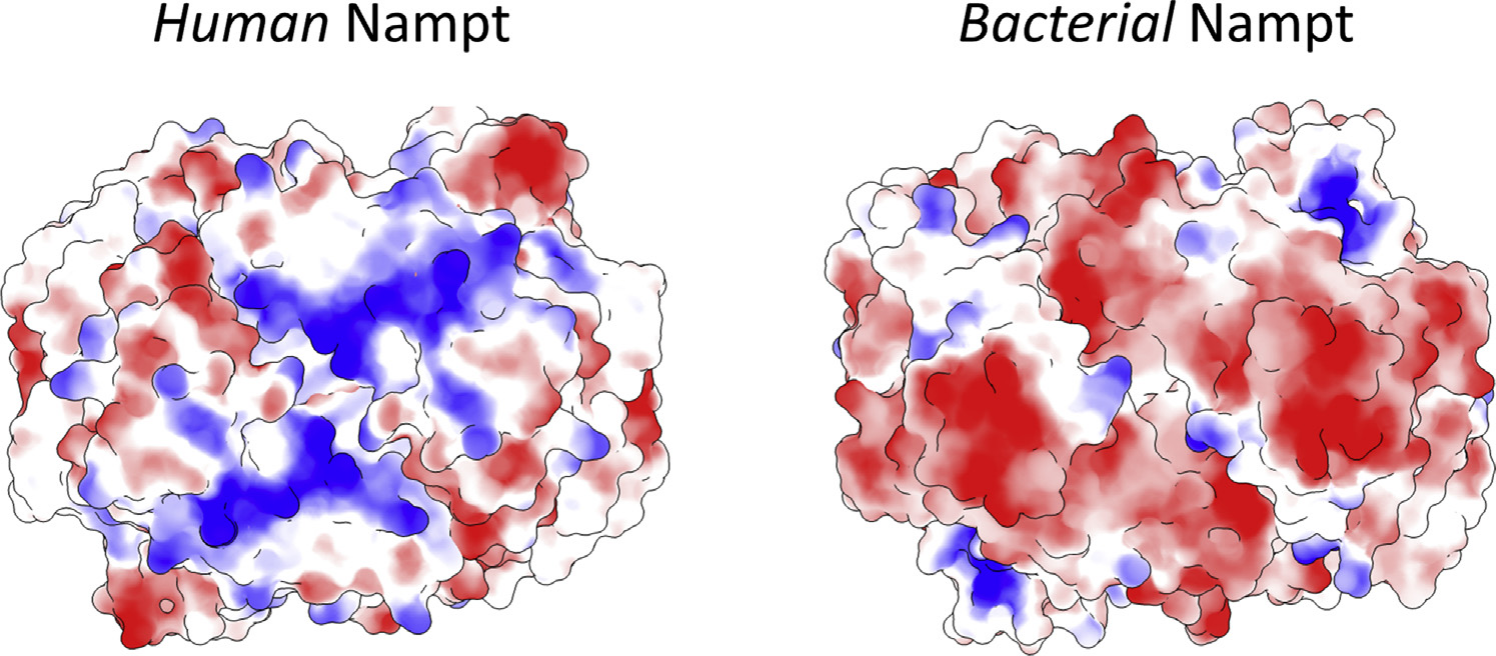
**Top view of human (PDB:****4o18****) and *Acinetobacter bayly* NadV (model) in surface representation.***Colors* represent electrostatic potential (*blue*, positive; *red*, negative).

Based on our previous finding that the bacterial ortholog of human NAMPT does not possess cytokine-like properties, as demonstrated by the inability of *Acinetobacter bayly* NAMPT (*Ab*NadV) to activate NF-κB signaling in macrophages (16), we performed a bioinformatic analysis in search of signature/s distinctive of the human enzyme that might be responsible for its signaling function. We first performed a multiple sequence alignment of NAMPT from evolutionary distant organisms, including mammals, lower eukaryotes, and bacteria (Fig. 1). The analysis revealed a lysine-rich sequence _41_EKKTENSK[R]XR[K]KV_52_ (where X is a bulky hydrophobic amino acid) connecting the β1 and β2 strands (hereafter named β1-β2 K-rich loop) in all NAMPTs from vertebrates. In NAMPTs from bacteria, including *Ab*NadV, such motif is usually absent or replaced by a shorter and nonconserved stretch of amino acids (Fig. 1). We then compared the human NAMPT with its bacterial ortholog at the structural level. In the absence of the 3D structure of the bacterial enzyme, we built a model of the *Ab*NadV dimer using the human ortholog as the template. As shown in Figure 2, a marked difference in the electrostatic potential distribution is noted between the human and bacterial enzymes. In particular, human NAMPT displays a large, X-shaped, positively charged region on the top of the dimer, absent in the bacterial ortholog. We found that a lysine-rich stretch (_68_KYLKGKVVTK_77_), encompassing a loop between α1 and α2 helices (hereafter named α1-α2 K-rich loop) and located about 20 residues downstream of the β1-β2 K-rich loop, is the main contributor to such positively charged area.

**Figure 3:**
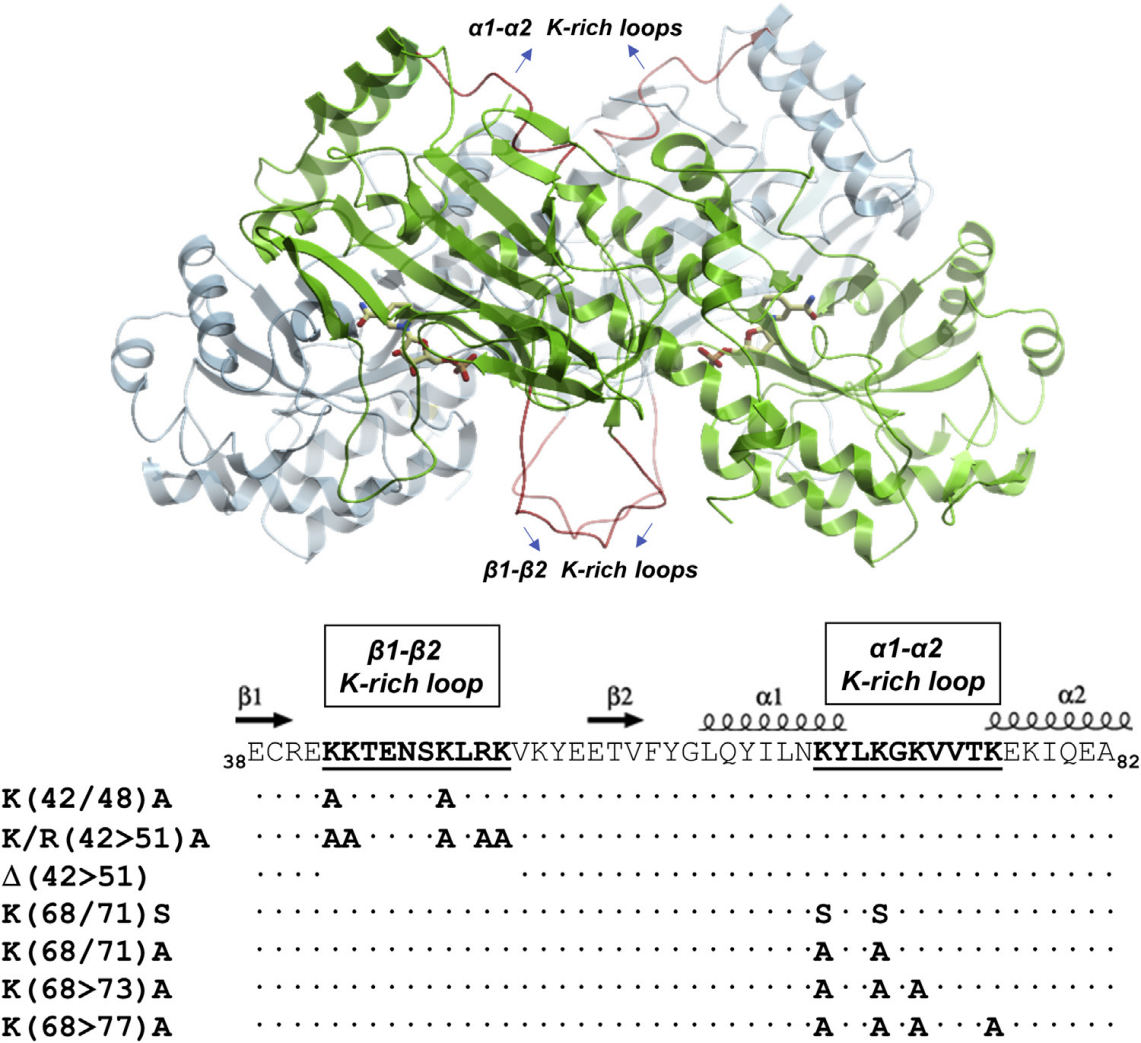
**Structure of human homodimeric NAMPT as *ribbon* diagram, with modeled, mutated loops highlighted in *red*.** Positions of active sites are shown with liganded NMN products (rendered as *sticks*). On the *bottom* the scheme of multiple mutants planned in this study is shown. Secondary structure elements of human NAMPT are represented by *spirals* (α helices) and *arrows* (β strands) and shown on the *top* of the alignment.

Altogether, these findings prompted us to select the β1-β2 and the α1-α2 K-rich loops as potential regions to be probed by site-directed mutagenesis for their interaction with TLR4. In Figure 3 the position of the identified loops in the context of human NAMPT dimer is shown.

### Molecular and kinetic characterization of NAMPT mutated proteins

To verify whether the β1-β2 and the α1-α2 K-rich loops were indeed involved in the signaling function of the enzyme, we generated the mutants shown in Figure 3. In selecting residues for mutagenesis, we focused on the positively charged amino acids for substitution, since such residues are commonly found to participate in protein–protein interactions, including the formation of TLR complexes (17, 18).

Regarding the β1-β2 K-rich loop, the entire set of positive residues or two lysine residues were replaced by alanine in mutants K/R(42>51)A (β-loop full mutant) and K(42/48)A (β-loop double mutant), respectively. In addition, the loop was fully deleted in the Δ(42>51) mutant (β-loop deletion mutant) (Fig. 3). As to the α1-α2 K-rich loop, mutants with two, three, or four lysine residues substituted with alanine were generated (Fig. 3). However, only the triple mutant K(68>73)A (α-loop triple mutant) was expressed in soluble form (Fig. S1), whereas both the double and the quadruple mutants were expressed as inclusion bodies (not shown), indicating that this K-rich region is important for the enzyme to maintain its conformation. The replacement of K68 and K71 with serine yielded a soluble K(68/71)S protein (α-loop double mutant) (Fig. S1).

As expected, the mutated proteins present a different extension of the positive patches when compared with the wildtype enzyme (Fig. S2).

The oligomeric state of the mutants was analyzed through size exclusion chromatography. When loaded onto the column at a concentration of 0.9 mg/ml, all mutated proteins exhibited a molecular weight of about 76,000, which is similar to the molecular weight of wildtype NAMPT determined under the same conditions, indicating that mutations did not affect the proteins' dimerization (Fig. S3).

Table 1 shows the kinetic properties of the mutated enzymes, as determined by using a previously reported sensitive fluorometric assay (19). As NAMPT is known to be markedly inhibited by the substrates (20), the effect of the mutations on substrate inhibition was also tested. For all mutants, except for the deleted protein, the apparent *K*_m_ for nicotinamide (Nam) was lower than the sensitivity threshold of the assay (*i.e.*, lower than 0.1 μM), so hindering comparison with the *K*_m_ of the wildtype enzyme, which is in the low nanomolar range (20). With regard to phosporibosyl pyrophosphate (PRPP), a *K*_m_ value of about 0.5 μM was determined for the wildtype enzyme, confirming previous results (20, 21). Both mutants in the α1-α2 K-rich loop retained the same catalytic efficiency of the wildtype enzyme, showing similar *K*_m_ and V_max_ values. Both mutants also exhibited inhibition by Nam and PRPP as the wildtype enzyme. These results are supported by the position of the α1-α2 K-rich loop, which is away from the active site (Fig. 3). The β-loop double and full mutants exhibited a slight decrease in PRPP substrate affinity. In addition, the β-loop full mutant showed a *K*_i_ value toward PRPP ∼20-fold higher than the wildtype, indicating a lower sensitivity to PRPP inhibition. Inspection of the 3D structure of human NAMPT shows that the β1-β2 K-rich loop is close to the PRPP-binding site, but its involvement in substrate binding cannot be anticipated owing to its unstructured conformation. Our kinetic results suggest that it has a marginal role in PRPP binding, whereas it is required for the PRPP inhibitory effect at high concentrations.

**Table 1:** Kinetic properties of the NAMPT proteins

NAMPT protein	*K*_m_ (μM)		V_max_ (nmol/min/mg)	V_max_/*K*_m_	*K*_i_ (μM)	
	Nam^a^	PRPP^b^	PRPP		Nam	PRPP
Wildtype	<0.1	0.46 ± 0.13	123 ± 2	586	103 ± 22	52 ± 13
α-Loop double mutant K(68/71)S	<0.1	0.28 ± 0.08	122 ± 14	381	93 ± 19	93 ± 24
α-Loop triple mutant K(68>73)A	<0.1	0.29 ± 0.05	108 ± 23	512	69 ± 19	104 ± 20
β-Loop double mutant K(42/48)A	<0.1	0.73 ± 0.14	148 ± 13	487	166 ± 41	103 ± 23
β-Loop full mutant K/R(42>51)A	<0.1	0.89 ± 0.14	110 ± 11	177	183 ± 52	905 ± 225
β-Loop deleted mutant ΔK(42>51)^c^	<0.1	0.12 ± 0.01	0.6 ± 0.02	5	ND	ND
	172	780 ± 40	1.3 ± 0.05	0.0017	ND	ND

Abbreviation: ND, not detectable.

a At 0.1 mM PRPP, 1 mM ATP.

b At 5 μM Nam, 1 mM ATP.

c Upper row shows kinetic parameters for the first phase (*i.e.*, K_m1_, V_max1_), lower row for the second stage of kinetics (*K*_m2_, V_max2_).

The kinetic behavior of the deleted protein was particularly striking. With both substrates, a biphasic Michaelis–Menten kinetics was observed (Fig. S4). In detail, at substrate concentrations in the low micromolar range, the affinity of the mutant toward the two substrates was comparable with that of the wildtype enzyme, although the velocity of the catalyzed reaction was significantly reduced. At higher substrate concentrations, the reaction velocity increased, although remaining much lower than that of the wildtype, and the affinity for both substrates significantly decreased. Fitting of the initial rates to the Michaelis–Menten equation (see Experimental procedures) yielded the apparent *K*_m1,2_ and *V*_max1_,_2_ values for the two phases reported in Table 1. To date, only a few examples of enzymes with a biphasic kinetic behavior have been reported (22, 23). In general, such behavior is indicative of the occurrence of multiple assembled states and/or multiple sites with different affinities for the substrates. It can be hypothesized that the deleted mutant exists in two different assembled states with distinct kinetic properties. The increase of the substrates might favor the transition from a high-affinity form to a lower-affinity form but with higher activity. The importance of the loop for a proper protein assembly is also evident from the finding that the activity of the mutant significantly depends on the protein concentration. In fact, its specific activity increased as a function of its concentration in the assay mixture, whereas the wildtype enzyme activity remained constant over the tested concentration range (Fig. S5). Another interesting feature of this mutant is its insensitivity to substrates' inhibition at the concentrations reported to inhibit the wildtype enzyme (Table 1).

### Kinetics of NAMPT proteins interaction with immobilized TLR4

**Figure 4:**
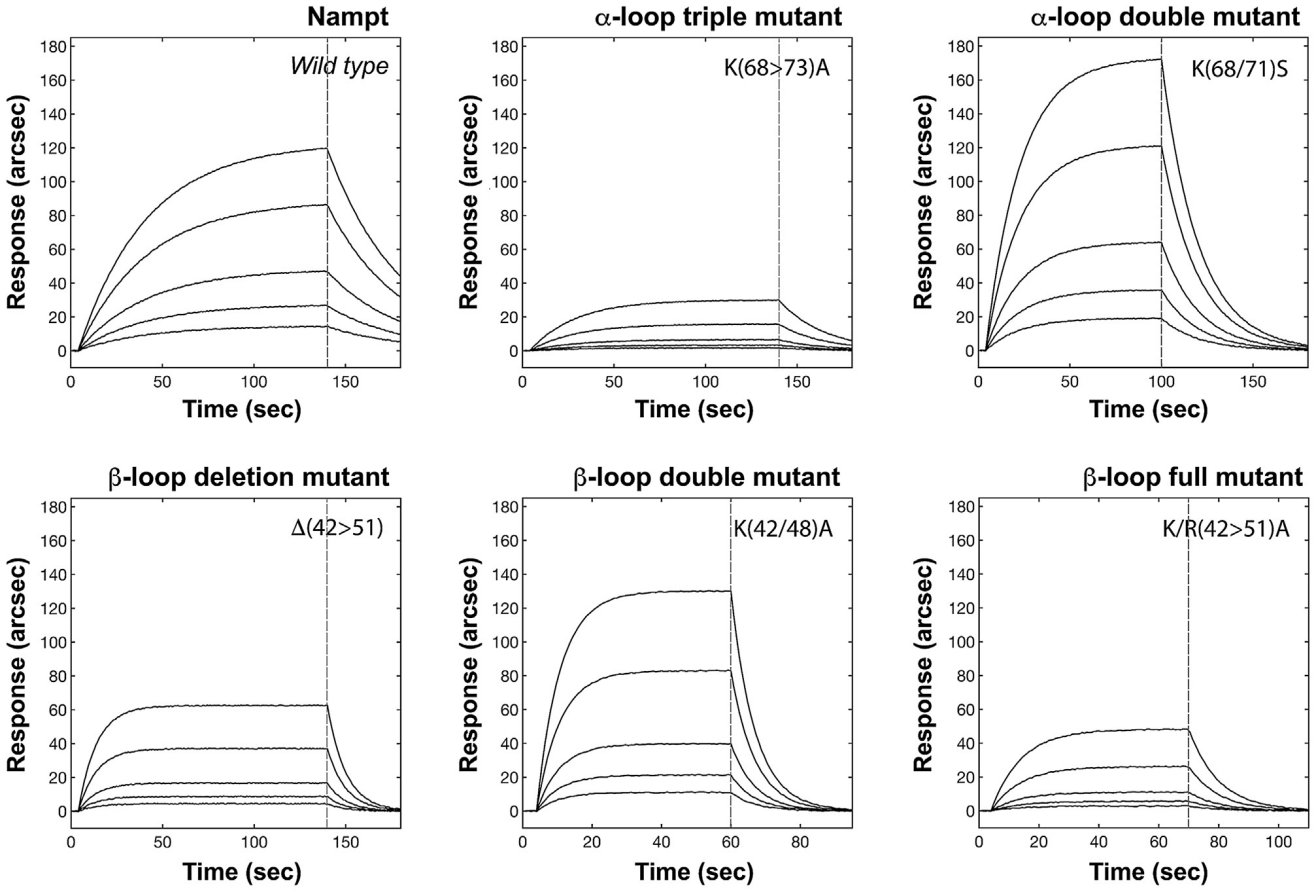
**Comparative superimposition of surface plasmon resonance sensorgrams independently obtained upon addition of increasing concentrations of NAMPT (either wildtype or mutants) in the range 1 to 3 nM to surface-blocked TLR4**.

The binding of NAMPT and mutated proteins to TLR4 was analyzed by surface plasmon resonance technology. NAMPT binds TLR4 in a concentration-dependent manner, with an equilibrium dissociation constant (K_D_) of about 18 nM (Fig. 4 and Table 2). All mutated enzymes retained the binding ability toward TLR4 (Fig. 4). Nevertheless, the comparative characterization of the interaction between individual NAMPTs and surface-blocked receptors revealed significant differences in the complex affinity both in terms of kinetics and thermodynamics of binding (Table 2). Specifically, the α-loop double mutant showed a K_D_ value (20.8 nM) similar to that of the wildtype protein, whereas the triple mutant exhibited a significantly higher K_D_ (1.28 μM), essentially owing to a 100-fold lower *k*_*ass*_ value. On the other hand, mutations in the β1-β2 K-rich loop did not affect the recognition phase (*k*_*ass*_), but they decrease to a different extent the kinetic stability of the complexes (*k*_*diss*_). In particular, substitution of K42 and K48 resulted only in a slightly higher K_D_ value (37 nM) when compared with the wild-type protein, whereas the substitution of all positive residues and the deletion of the loop significantly decreased the affinity toward TLR4 with K_D_ values of 0.63 and 0.22 μM, respectively. The fitting of raw kinetic data revealed a monoexponential behavior, suggesting the presence of a single high-affinity binding site for NAMPT(s) on TLR4 (the biexponential model did not significantly improve the quality of the fit as judged by an F-test, 95% confidence).

**Table 2:** Kinetic and equilibrium parameters of the interactions between surface-blocked TLR4 and soluble NAMPT variants

TLR4-NAMPT complex	*k*_*ass*_ (M^−1^s^−1^)	*k*_*diss*_ (s^−1^)	*K*_*D*_ (M)
TLR4-wild type	(2.8 ± 0.3) 10^5^	0.005 ± 0.001	(1.78 ± 0.39) 10^−8^
TLR4–α-loop double mutant K(68/71)S	(4.8 ± 0.7) 10^5^	0.01 ± 0.004	(2.08 ± 0.89) 10^−8^
TLR4–α-loop triple mutant K(68>73)A	(3.1 ± 0.7) 10^3^	0.004 ± 0.002	(1.28 ± 0.70) 10^−6^
TLR4–β-loop double mutant K(42/48)A	(7.8 ± 0.5) 10^5^	0.029 ± 0.003	(3.71 ± 0.46) 10^−8^
TLR4–β-loop full mutant K/R(42>51)A	(1.3 ± 0.7) 10^5^	0.085 ± 0.023	(6.30 ± 3.50) 10^−7^
TLR4–β-loop deleted mutant Δ(42>51)	(3.2 ± 0.4) 10^5^	0.07 ± 0.02	(2.18 ± 0.68) 10^−7^

### Effect of NAMPT proteins on NF-κB signaling in cultured cells

Based on our previous evidence that treatment of human-derived macrophages with NAMPT induced a TLR4-dependent robust activation of NF-κB signaling (16), we investigated in these cellular models the ability of the NAMPT mutated proteins to trigger the NF-κB pathway. In particular, phosphorylation of the p65 subunit and *IL1B* transcription driven by exposure to the α-loop triple mutant and the β-loop full mutant were compared with those driven by the wildtype (WT) protein.

**Figure 5:**
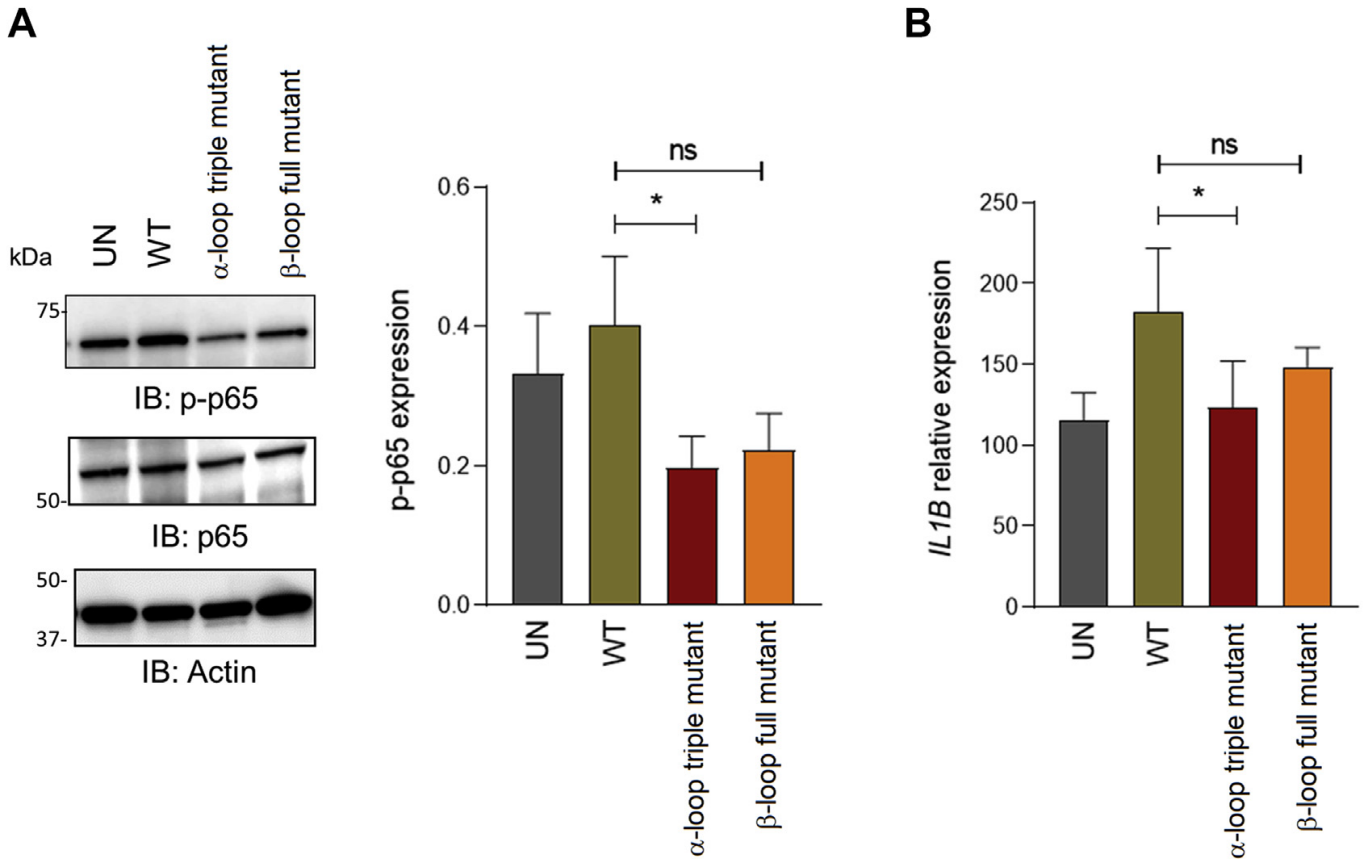
**NAMPT mutants reduce the activation of NF-κB pathway.***A*, representative Western blot analysis of phospho(p)-p65 in healthy donor macrophages (n = 5) upon treatment (20 min) with wildtype NAMPT (WT) or the α-loop triple mutant K(68>73)A and β-loop full mutant K/R(42>51)A (100 ng/ml, 20 min). On the *right*, the histogram represents cumulative densitometric analysis of p-p65 in five different preparations of macrophages. Actin was used as loading control. *B*, histogram showing mRNA expression levels of *IL1B* in macrophages (n = 5) treated with indicated NAMPT variants (100 ng/ml, 6 h). Data were expressed as mean ± SEM. Paired *t* test was used to calculate statistical significance. ∗ *p*< 0.05, ns: not significant. UN, untreated condition.

Through a dose–response experiment we selected 100 ng/ml as the optimal NAMPT concentration to induce p65 phosphorylation (Fig. S6), in agreement with previous studies (2, 14, 16). As shown in Figure 5 and Fig. S7, a significant reduction in both p65 phosphorylation and *IL1B* mRNA levels was observed in cells treated with the α-loop triple mutant compared with wildtype (100 ng/ml, 20 min for protein phosphorylation and 6 h for gene expression). Although not statistically significant, a reduction in phosphorylated p65 was also observed with the β-loop full mutant. We observed a decrease in p65 phosphorylation levels in mutants-treated cells when compared with untreated cells (Figs. 5*A* and S7). This might be related to the occupancy of the TLR4 receptor by inert proteins that would prevent any basal signaling. This effect was not observed when considering the modulation of downstream gene expression, *i.e.*, *IL1B* transcription (Fig. 5*B*). Altogether, these results confirm that the mutations impairing TLR4 binding *in vitro* also affect the NAMPT ability to prime signaling in human macrophages.

Activation of TLR4 by the bacterial cell wall component lipopolysaccharide (LPS) that triggers the innate immune response to pathogens requires two coreceptors, *i.e.,* the adaptor protein myeloid differentiation factor 2 (MD2) and CD14 (24). We took advantage of an engineered human TLR4 reporter cell model optimized for the LPS-induced signaling to examine the effect of NAMPT and mutated proteins on TLR4 activation in the presence of MD2 and CD14. The used cells (HEK-Blue hTLR4) are stably transfected to coexpress high levels of TLR4, MD2, and CD14 for LPS recognition and binding. TLR4 stimulation is assessed by measuring the activity of NF-κB-dependent secreted alkaline phosphatase (SEAP). Neither NAMPT nor the mutated proteins stimulated the production of SEAP, indicating that NAMPT is not able to bind TLR4 when it is complexed to MD2 (not shown). Besides, these results also ruled out the presence of significant levels of endotoxin contaminations in the protein preparations. In fact, by referring to a standard curve obtained with LPS, we determined LPS levels lower than 2 pg LPS (0.001 endotoxin units)/μg protein in all preparations (Fig. S8).

### Molecular docking studies on NAMPT–TLR4 interaction

**Figure 6:**
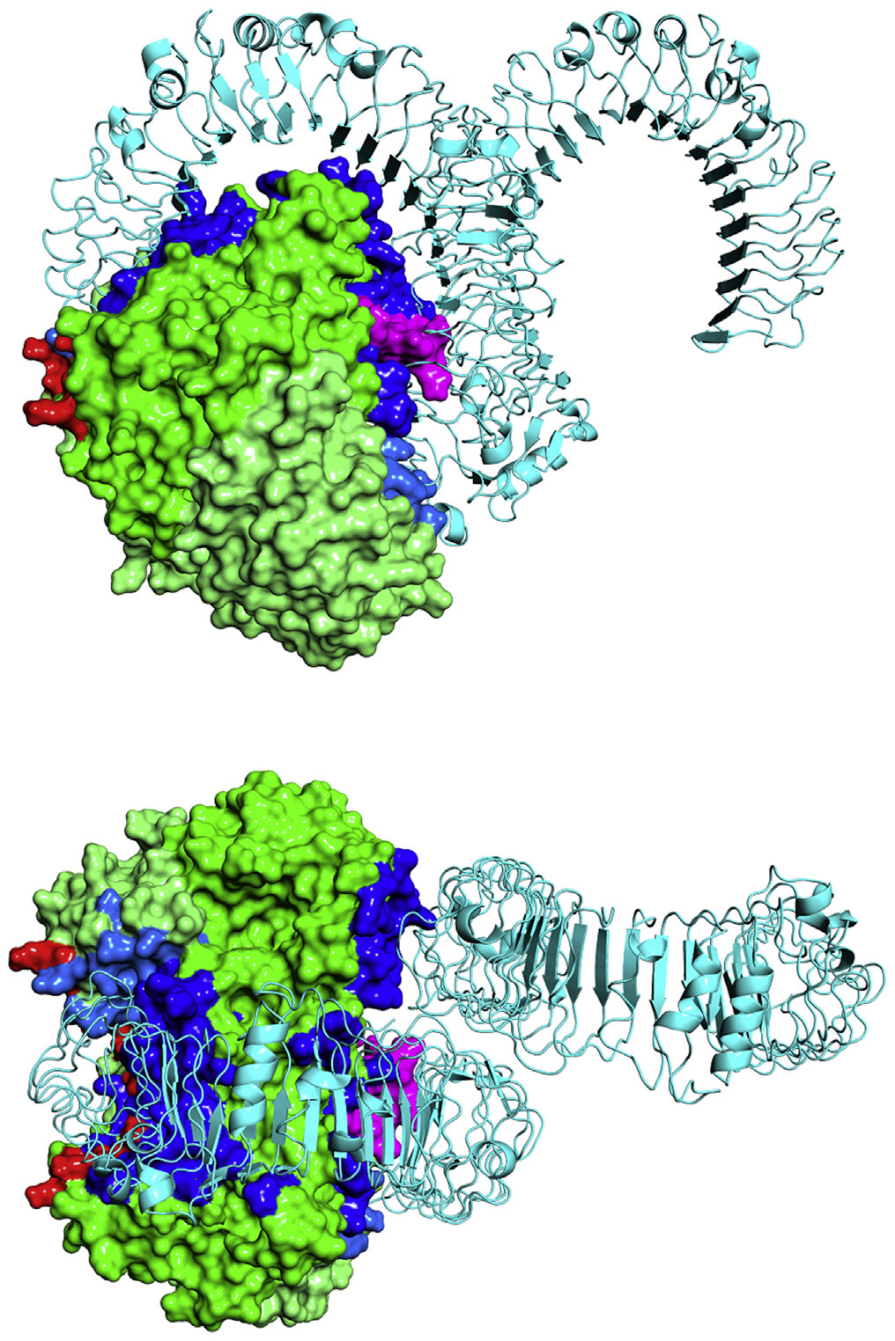
**Front (*upper panel*) and top view (*lower panel*) of the NAMPT–TLR4 complex obtained by docking human NAMPT dimer (monomers are rendered as *green* and *light green solid surfaces*) onto homology modeled TLR4 (*gray ribbon*).** NAMPT amino acids of the two monomers involved in the interaction with TLR4 are rendered as *dark* and *light blue* surfaces, respectively. α1-α2 and β1-β2 K-rich loops on NAMPT are highlighted in *red* and *purple*, respectively.

To further gain insights into NAMPT–TLR4 interaction we performed molecular docking between TLR4 homodimer and NAMPT homodimer. TLR4 cellular function is known to be exerted upon the receptor dimerization (25). On the other side, NAMPT presents a stable homodimeric quaternary structure, as already clarified by other authors (26, 27, 28) and by this work (see gel filtration data). The minimized model for the NAMPT–TLR4 complex (Fig. 6) confirms the role of the NAMPT-positive patches, showing the presence of stabilizing electrostatic interactions between Lys residues 48, 68, 71, 73 and TLR4 negatively charged carboxylic moieties (Table S1).

## Discussion

Previous studies, also from our group, established that, in some cell types, TLR4 is required for the DAMP activity of eNAMPT (14, 16). A direct interaction between human recombinant NAMPT and the extracellular domain of TLR4 was demonstrated through SPR analyses by coating an NAMPT antibody on the sensor chip and showing that a premixed solution of NAMPT and TLR4 resulted in increasing binding when compared with NAMPT alone (14, 16). Here, we have analyzed the kinetics of the NAMPT–TLR4 complex formation by assessing the direct binding of NAMPT to immobilized TLR4. We measured a high binding affinity, with a K_D_ value of about 18 nM, very similar to that of LPS, the first validated natural ligand of TLR4, which shows K_D_ values ranging from 3 to 10 nM (24, 29). The binding affinity of NAMPT to the receptor is also very similar to that of other DAMPs reported to trigger TLR4, like peroxiredoxin-5 (30) and wheat amylase/trypsin inhibitors (31). Also, NAMPT shows an affinity toward TLR4 higher than high mobility group box 1 protein (HMGB1) that binds the receptor with a K_D_ of about 0.6 μM (32).

To shed light on the molecular NAMPT–TLR4 interaction, we started from the evidence that bacterial NAMPT does not act as a cytokine (16). Therefore, we sought molecular determinants that might be responsible for the interaction among the distinctive structural elements of the human enzyme. By combining bioinformatics, mutagenesis, and SPR analyses, we identified two surface loops involved in the interaction with the receptor, referred as to β1-β2 and α1-α2 K-rich loops. Mutation of the positively charged residues within the two loops not only affected the binding of the protein to the receptor in SPR experiments but also impaired NAMPT's ability to trigger the NF-κB pathway in cultured human macrophages.

A recent study showed that the β1-β2 loop has been acquired by NAMPT in higher vertebrates and demonstrated that such acquisition would have given the enzyme an increased catalytic efficiency (33). Our kinetic analyses indicated that the loop is also required to maintain the enzyme in a properly assembled form, as its deletion not only reduces the catalytic activity but also induces an allosteric response of the enzyme to both substrates. Besides, replacing all the loop's positively charged residues with alanine significantly lowers the enzyme's sensitivity toward PRPP substrate inhibition. Strikingly, in all the 66 protein structures currently deposited in the Protein Data Bank database for human NAMPT, the central region _44_TENSKLRK_51_ of the loop has evaded structure determination, which is indicative of a highly mobile loop, likely involved in the interaction with other molecules. In keeping with this, the SPR analysis clearly showed its involvement in TLR4 binding. Indeed, both its deletion and the substitution of all lysine and arginine to alanine caused a faster dissociation of NAMPT from the receptor. This same loop has been recently demonstrated to be involved in the interaction of murine NAMPT with GAPDH, which is responsible for translocating NAMPT from the cytosol to the nucleus to sustain nuclear NAD replenishment under stress conditions (34).

The α1-α2 loop forms a positively charged area on the top of the NAMPT dimer. The SPR analysis has clearly indicated that the lysine residues in such area are involved in the protein interaction with TLR4, as the simultaneous replacement of three residues with alanine markedly weakened the association to the receptor. We have previously reported that the enzyme nicotinate phosphoribosyltransferase (NAPRT), which catalyzes the first step in the biosynthesis of NAD starting from nicotinic acid, shares with NAMPT the ability to act as an extracellular mediator of inflammation. Indeed, elevated circulating NAPRT can be measured in patients with sepsis and septic shock (16). Like NAMPT, human NAPRT triggers the TLR4-dependent NF-κB pathway in human macrophages and shows a positively charged region involved in the signaling function, which is similarly positioned as the NAMPT α1-α2 loop (16). For both NAMPT and NAPRT, the positive patches involved in TLR4 binding are not conserved in the bacterial counterparts, which might explain why the bacterial proteins do not behave as cytokines (16). By acquiring positive patches on their surface, the two enzymes would have gained the ability to interact with the receptor, thus becoming extracellular mediators of inflammation. Several studies report that positive patches on protein surfaces can indicate binding to nucleic acids, membranes, ligands, or proteins (35, 36).

The molecular mechanism of TLR4 activation by DAMPs is poorly investigated. Fibronectin requires the same coreceptors and accessory molecules as LPS to trigger the TLR4-dependent signaling, whereas other DAMPs, like hyaluronan and the tenascin C protein, require neither MD2 nor CD14 for TLR4 activation (37, 38). Our result that NAMPT is not able to activate TLR4 in a cell model engineered to respond to LPS by coexpressing TLR4, MD2, and CD14 strongly suggests that the canonical TLR4/MD2/CD14 receptor complex is not involved in the eNAMPT/TLR4 signaling. This is in keeping with both the SPR results, showing that the protein strongly binds to the receptor in the absence of MD2, and the docking results, revealing that NAMPT interacts with TLR4 in its concave surface, similarly to MD2 (39). Owing to the steric hindrance, competitive binding of NAMPT and MD2 to TLR4 can be envisioned to occur *in vivo*. In this view, NAMPT/TLR4 signaling might be of relevance in those cells, like airway or corneal epithelial cells, which have limited response to LPS due to a low or absent expression of MD2 (40, 41). Further work is required to determine whether TLR4 activation by NAMPT relies on accessory molecules and/or coreceptors in specific cell types.

In conclusion, our work identified NAMPT structural determinants involved in TLR4 binding as positively charged patches acquired by the vertebrate enzyme during evolution. Their targeting might represent a promising therapeutic strategy against the eNAMPT-induced inflammatory response.

## Experimental procedures

### Site-directed mutagenesis

Site-directed mutagenesis was carried out using the QuickChange Lightning kit (Agilent Technologies). The deleted mutant was obtained using the In-Fusion HD Cloning Plus kit (Taqara Bio). Both kits were used according to manufacturer's instructions with minor variations. The plasmid pET15-b harboring the wildtype *NAMPT* gene (42) was used as a template for PCR mutagenic reactions. The sequence of mutagenic primers is shown in Table S2. For amino acids replacement, PCR was set up using about 20 ng of plasmid and 125 ng of mutagenic primers. After the temperature cycling step, the parental template was digested by DpnI. The resulting mutant DNA was concentrated by ethanol precipitation. The pellet was dried, resuspended in 5 μl of sterile water and used to transform XL10-Gold ultracompetent cells, according to the instructions. For the deleted mutant, PCR was carried out by using 10 ng of template DNA, the appropriate mutagenesis primers, and the Clone Amp HiFi PCR premix, according to the supplier's protocol. About 100 ng of amplified mutant DNA, purified by gel extraction, was used in 10 μl of In-Fusion reaction, and 2.5 ul of the mix was used to transform competent cells supplied by the kit. All mutants were sequenced to verify incorporation of the desired modification and to ensure the absence of random mutations.

### Preparation of recombinant proteins

Both the pET15b plasmid harboring wildtype *NAMPT* (42) and the mutagenized plasmids were transformed into *ClearColi BL21* (DE3) cells (Lucigen). All proteins were expressed and purified by Ni-NTA affinity chromatography as described (42). Active fractions were passed through a PD10 column equilibrated and eluted with 50 mM Hepes, pH 7.5, 0.3 M NaCl, 20% glycerol. The protein preparations were stored at −20 °C for further analyses. To ensure removal of endotoxin contamination for biosensor and cellular experiments, protein preparations were treated with ε-poly-L-lysine resin (Thermo Scientific) that binds LPS with high affinity. In detail, the resin was equilibrated with endotoxin-free buffer consisting of 50 mM Hepes, pH 7.5, 0.3 M NaCl (for cellular experiments) or 50 mM sodium phosphate, pH 7.5, 0.15 M NaCl (for biosensor analyses). The protein sample was incubated with the equilibrated resin with gentle mixing for 1 h at 4 °C. The resin was removed by centrifugation at 500*g* for 1 min.

TLR4 was expressed and purified as reported elsewhere (31). Briefly, the plasmid pEFBOS-TLR4 (kindly provided by Dr Kensuke Miyake, University of Tokyo) was transfected in HCT-116 cells. Transfected cells were lysed by syringe passage in 20 mM Mops pH 7.0, 0.3 M NaCl, 5 mM imidazole, 5% (v/v) glycerol, 10 mM β-mercaptoethanol, 0.3% (v/v) Triton X-100, 0.5 mM PMSF. The cell homogenate was centrifuged at 15,000*g* for 30 min at 4 °C, discarding the pellet. TLR4 was purified through immobilized metal-ion affinity chromatography and gel filtration chromatography. The purified protein was freeze-dried and stored at −80 °C until use.

### Gel filtration chromatography

Gel filtration chromatography was carried out to assess the correct folding of mutated proteins, comparing them with wildtype NAMPT. Proteins were loaded onto a Superose 12 10/300 GL column (GE Healthcare) and eluted with 50 mM Hepes, pH 7.5, 0.3 M NaCl. Standard proteins were bovine serum albumin (66 kDa), ovalbumin (45 kDa), and carbonic anhydrase (30 kDa).

### Kinetic analyses

The catalytic activity of NAMPT and mutated proteins was determined by using the highly sensitive fluorometric assay described in (19). In detail, in a first reaction mixture (210 μl), the NAMPT-catalyzed NMN formation was coupled to NAAD production, by incubating appropriate amounts of enzymes in 70 mM Hepes, pH 7.5, 10 mM MgCl_2_, 2.5 mM ATP, 0.6 U/ml purified recombinant *B. anthracis* NadD, 0.15 U/ml purified recombinant *E. coli* PncC, PRPP, and Nam at the indicated concentrations. At different times, 60-μl aliquots were withdrawn, and the reaction was stopped by heating at 95 °C for 1 min. In a subsequent step, the produced NAAD was stoichiometrically converted to NAD by incubation of 40 μl of the heated sample in 50 mM Hepes, pH 7.5, 0.15 M KCl, 1.4 mM ATP, 11 mM MgCl_2_, 50 mM NH_4_Cl, and 0.06 U/ml purified recombinant *B. anthracis* NadE (145 μl). After 30 min at 37 °C, the formed NAD was cycled and quantitated as described (19). One unit of NAMPT is defined as the amount of enzyme that catalyzes the formation of 1 μmol NMN per minute, at 37 °C.

*K*_m_, V_max_, and *K*_i_ values were calculated by fitting initial rates to a modified version of the Michaelis–Menten equation that takes into account the binding of a second, inhibitory molecule of substrate above a critical substrate concentration:

$$\text{V}={\text{V}}_{\text{max}}/\left(1+K_{\text{m}}/\left[\text{S}\right]+\left[\text{S}\right]/K_{\text{i}}\right)$$

where the term *K*_i_ represents the dissociation constant for the inhibitory SES ternary complex. Initial rates were fitted to the model using the software Prism 6 (GraphPad). For the kinetics of the Δ (42>51) mutant, showing a biphasic kinetic behavior, we used a modified Michaelis–Menten equation reflecting a mixture of two enzyme forms with different affinities for the same substrates:

$$\text{V}={\text{V}}_{\text{max}1}\text{·}\left[\text{S}\right]/\left(K_{\text{m}1}+\left[\text{S}\right]\right)+{\text{V}}_{\text{max}2}\text{·}\left[\text{S}\right]/\left(K_{\text{m}2}+\left[\text{S}\right]\right)$$

### Biosensor binding studies

Binding experiments were performed on an evanescent wave/resonant mirror optical biosensor (IAsys plus - Affinity Sensors Ltd), equipped with dual-well carboxylate cuvettes (NeoSensors, Ltd). TLR4-tethered sensing surfaces were prepared as described (31). Briefly, upon activation of carboxylate groups with an equimolar solution of EDC and NHS, TLR4 was covalently anchored *via* the N terminus of the histidine tail. A TLR4 concentration of 400 μg/ml was always used during surfaces preparation as it ruled out hindering effects that could reduce the number of available binding sites on the sensing surface (31). Specifically, instrumental response upon TLR4 immobilization indicated the coupling of a partial monolayer (surface density 1.3 ng/mm^2^, approximately equivalent to 10 mg/ml). Free carboxylic sites on the surface were inactivated with 1 M ethanolamine pH 8.5, and finally the surface was equilibrated with PBS. The absence of negative baseline drift signals with time or multiple PBS washes confirmed that the receptor molecules were covalently linked to the sensor surface. Sensing chamber was thermostatted at 37 °C throughout.

NAMPT and mutated proteins were tested for binding to surfaced-blocked TLR4 at different concentrations of the enzyme(s) in the range 1 to 3 nM. Response kinetics were routinely followed up to equilibrium, and baseline recovery was always assessed prior to any new analysis (the dissociation of TLR4–enzyme complexes was carried out by serial PBS washes). Raw data were locally and globally analyzed according to monoexponential and biexponential models (43).

### Human macrophages generation and treatment

Buffy coats from healthy donors to isolate peripheral blood mononuclear cells were obtained from the local Blood Bank. Peripheral blood mononuclear cells were seeded in 24-well plates (10^7^ per well) in monocyte attachment medium (1 h, 37 °C, PromoCell-GmbH). Nonadherent cells were removed before adding RPMI+10% fetal calf serum (Sigma-Aldrich) supplemented with recombinant human macrophage colony-stimulating factor (M-CSF; 50 ng/ml PeproTech) for 5 to 6 days (16). Fully differentiated macrophages were treated, for the indicated time, with wildtype or mutant NAMPTs (100 ng/ml).

### RNA extraction and quantitative real-time PCR

Quantitative real-time PCR was performed as described (16). TaqMan Gene Expression Assays (Thermo Fisher, Monza.IT) used Hs_01555410_m1 (*IL1B*) and Hs_00984230_m1 (*B2M*) as housekeeping gene.

### Western blot

Cells lysates were resolved by SDS-PAGE and transferred to nitrocellulose membranes (Bio-Rad) (16). Western blot reactions were visualized using ChemiDoc Touch Imaging System (Bio-Rad). Densitometric analyses were performed using Image Lab 6.0.1 Software (Bio-Rad). Band intensity was quantified after normalizing over the corresponding unphosphorylated protein or over actin, used as loading control.

### Effect of NAMPT on NF-κB signaling in HEK-Blue hTLR4 cells

HEK-Blue hTLR4 cells (InvivoGen) stably express human TLR4 with its MD2/CD14 coreceptors and an NF-κB-inducible secreted embryonic alkaline phosphatase (SEAP) reporter gene. Cells were grown in Dulbecco's modified Eagle's medium supplemented with 10% FBS, 2 mM L-glutamine, 100 μg/ml normocin, 50 U/ml penicillin, 50 g/ml streptomycin at 37 °C, with 5% CO_2_ and passaged when 70% confluence was reached. Cells were seeded in a 96-well flat-bottom plate and incubated for 24 h with different amounts of wildtype or mutated proteins (0.45–0.9 μM) or LPS (1–4 nM) to provide a calibration curve. At the end of incubation, the medium was replaced with HEK-Blue Detection medium, and after 16 h incubation at 37 °C, the activity of secreted SEAP was determined by measuring the absorbance at 620 nm.

### Sequences analysis

A structure-driven multiple sequence alignment of NAMPTs from 50 diverse eukaryotes (including lower unicellular organisms) and bacteria was obtained using PROMALS3D (44). The figure was produced with ESPript (45).

### Molecular modeling

Models (as homodimers) for the mutants of human NAMPT (P43490) studied in this work were obtained by homology modeling (46) using human NAMPT available X-ray structure (4kfn.pdb) as template. Model (as homodimer) for human NAMPT (P43490) and for *A. bayly* NadV were obtained from the Swiss-model repository (47). Steric clashes in the models were further minimized using short discrete molecular dynamics simulations (48). Model for the extracellular domain of the human TLR4 (AAY82270.1) was obtained by homology modeling (46) using a high-resolution mutant TLR4 available X-ray structure (4g8a.pdb) as template.

Poisson–Boltzmann electrostatic potential maps (49) have been calculated for NAMPT and mutants models using AMBER as force field, pH=7.0, linear solver for solving Poisson–Boltzmann equation, dielectric constants set to 4.0 (internal) and 80.0 (external), salt concentration equals to 0.15 M.

All the models have been prepared for docking adding proper hydrogens at pH=7.0 using Discovery studio software suite (Accelrys Inc). The wildtype NAMPT homodimer has been docked to TLR4 (homodimer) using the fast Fourier transform–based protein docking ZDOCK module (50). The best pose has been then further minimized using the DISCOVER module (CVFF force field, conjugate gradients minimizer, no restraints [flexible docking], maximum number of iterations equals to 50,000, convergence value as maximum derivative of 0.5 kcal mol^−1^ Å^−1^). The binding affinity of the NAMPT (homodimer)–TLR4 (homodimer) complex has been computed using interfacial contacts and noninteracting surfaces of the protein–protein complex (51).

## Data availability

The authors declare that all data supporting the findings of this study are contained within the article and its supporting information file. The model of *Ab*NadV can be found at https://swissmodel.expasy.org/interactive/VKU7PM/models/.

## Supporting information

This article contains supporting information.

## Conflict of interest

The authors declare that they have no conflicts of interest with the contents of this article.
