# Supplementary material for: Molecular insights into the interaction between human nicotinamide phosphoribosyltransferase and Toll-like receptor 4
Source: J Biol Chem. 2022 Feb 2;298(3):101669. doi: 10.1016/j.jbc.2022.101669 (PMC8892085; doi:10.1016/j.jbc.2022.101669)
Supplement: Supplemental Figures S1–S8, Tables S1 and S2 [file mmc1.docx]

**MOLECULAR INSIGHTS INTO THE INTERACTION BETWEEN HUMAN NICOTINAMIDE PHOSHPHORIBOSYLTRANSFERASE AND TOLL-LIKE RECEPTOR 4**

**Massimiliano Gasparrini^1$^, Francesca Mazzola^2$^, Massimiliano Cuccioloni^3^, Leonardo Sorci^4^, Valentina Audrito^5^, Federica Zamporlini^1^, Carlo Fortunato^1^, Adolfo Amici^2^, Michele Cianci^1^, Silvia Deaglio^5^, Mauro Angeletti^3^, Nadia Raffaelli^1*^**

^1^Department of Agricultural, Food and Environmental Sciences, Polytechnic University of Marche, Ancona, Italy

^2^Department of Clinical Sciences, Polytechnic University of Marche, Ancona, Italy

^3^School of Biosciences and Veterinary Medicine, University of Camerino, Camerino, Italy

^4^Department of Materials, Environmental Sciences and Urban Planning, Division of Bioinformatics and Biochemistry, Polytechnic University of Marche, Ancona, Italy

^5^Department of Medical Sciences, University of Turin, Turin, Italy

^$^equally contributed

*corresponding author

**
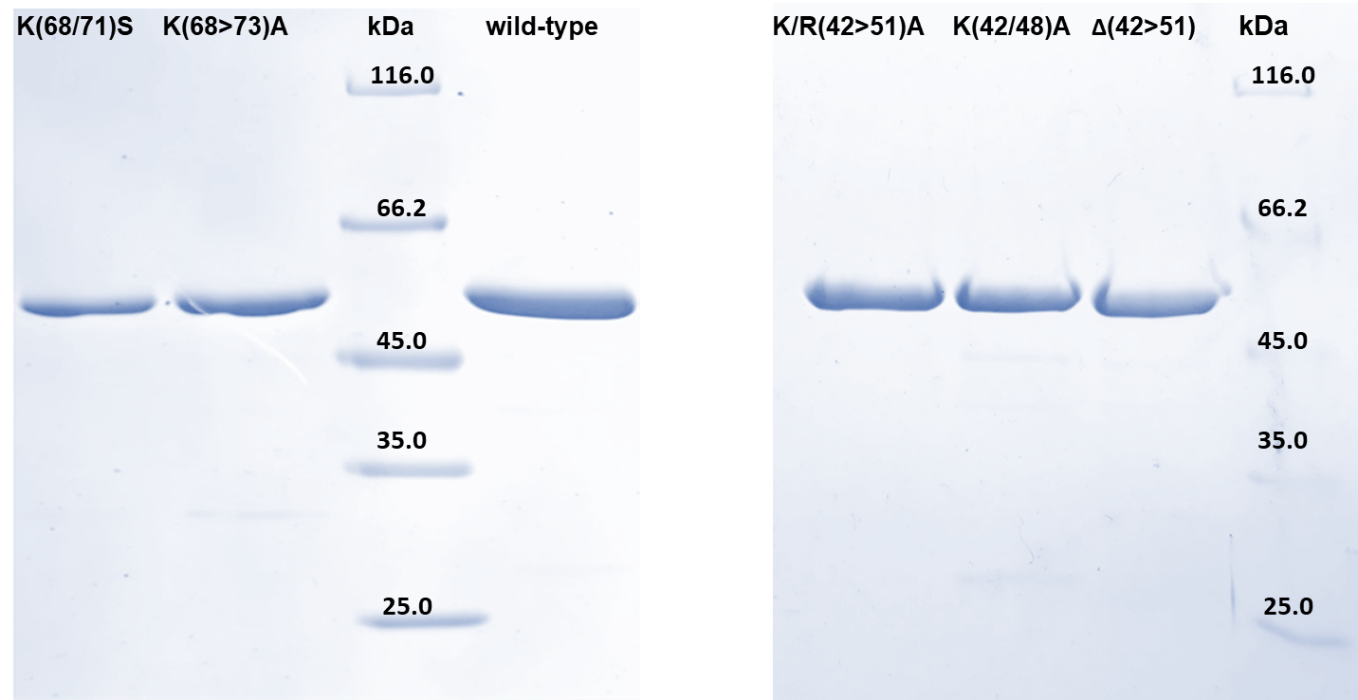
**

**Figure S1.** 12% SDS-PAGE of purified mutated proteins and wild-type NAMPT

**
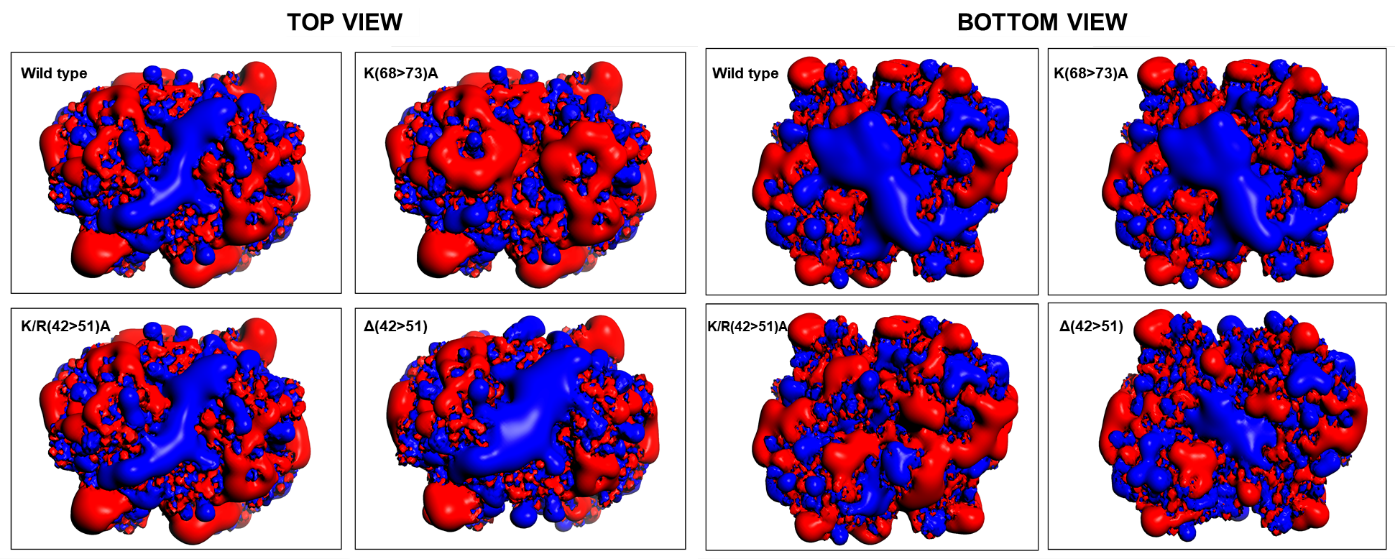
**

**Figure S2.** Bottom and top view of wild type NAMPT and selected mutated proteins in surface representation. Colours represent electrostatic potential (blue, positive; red, negative). Models of mutated NAMPTs were built as described in Experimental Procedures.

**
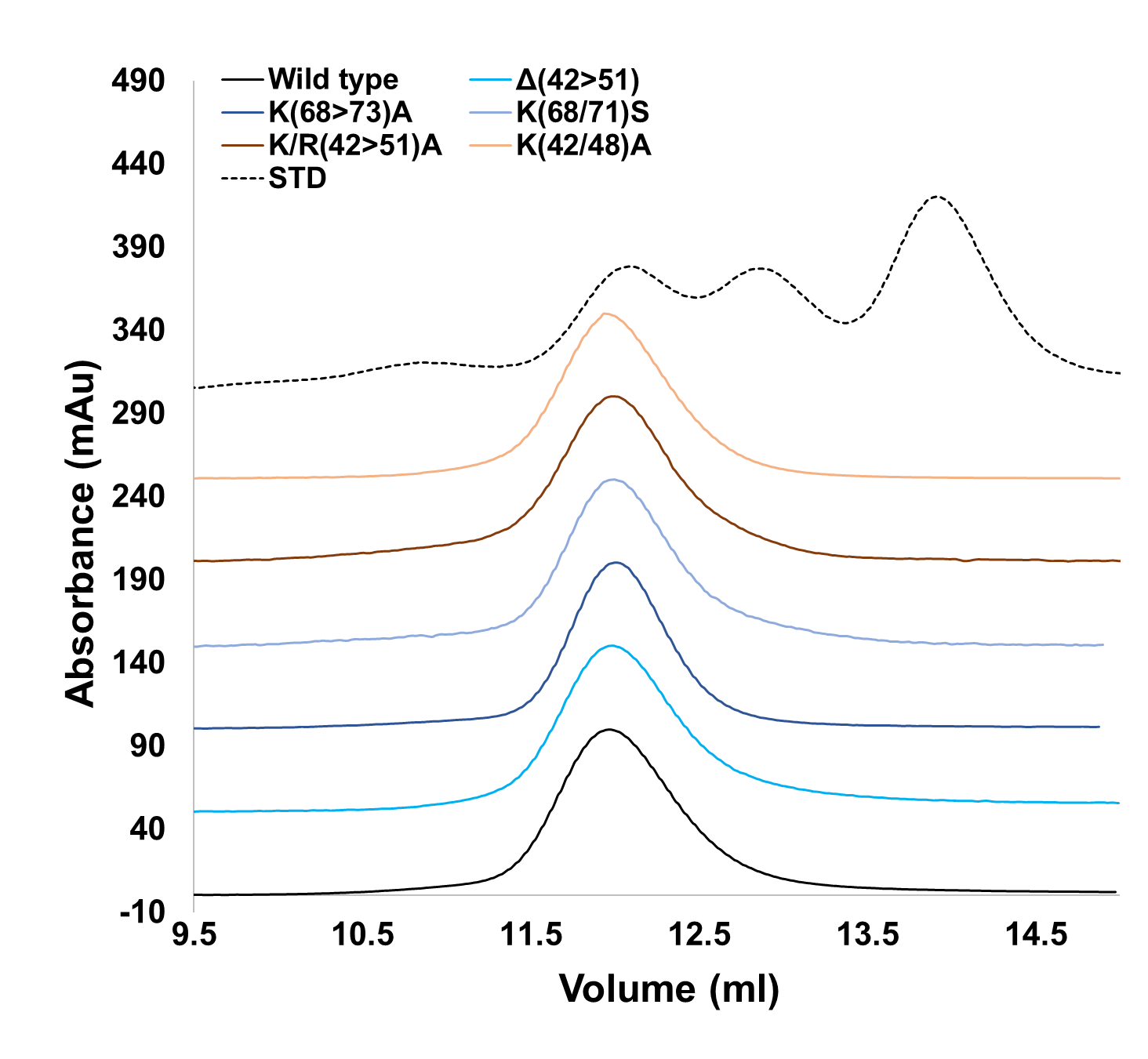
**

**Figure S3.** Elution profiles from gel filtration chromatography of wild-type NAMPT and mutated proteins. Elution of protein standards (a, bovine serum albumin dimer 145 kDa, b, bovine serum albumin monomer 66 kDa; c, ovalbumin 45 kDa; d, carbonic anhydrase, 20 kDa) is shown in dashed line.

**Figure S4.** Activity of the deleted NAMPT mutant as a function of substrates concentrations. (A) Nam ranged from 0.15 to 200 µM, at fixed 0.1 mM PRPP; (B) PRPP ranged from 0.125 to 1000 µM, at fixed 5 µM Nam.

**
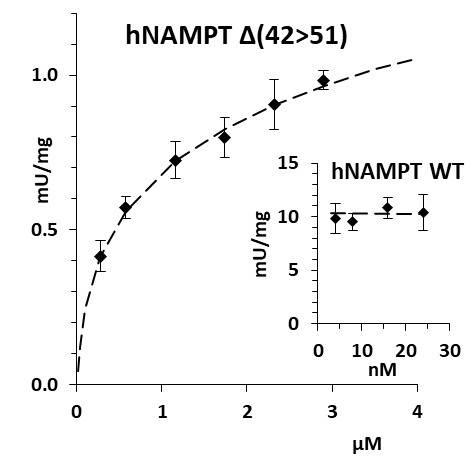
**

**Figure S5.** The specific activity of the deleted mutant and the wild-type enzyme (in the inset) as a function of the protein concentration in the assay mixture. The catalytic activity was assayed in the presence of 1 mM PRPP and 50 μM Nam.


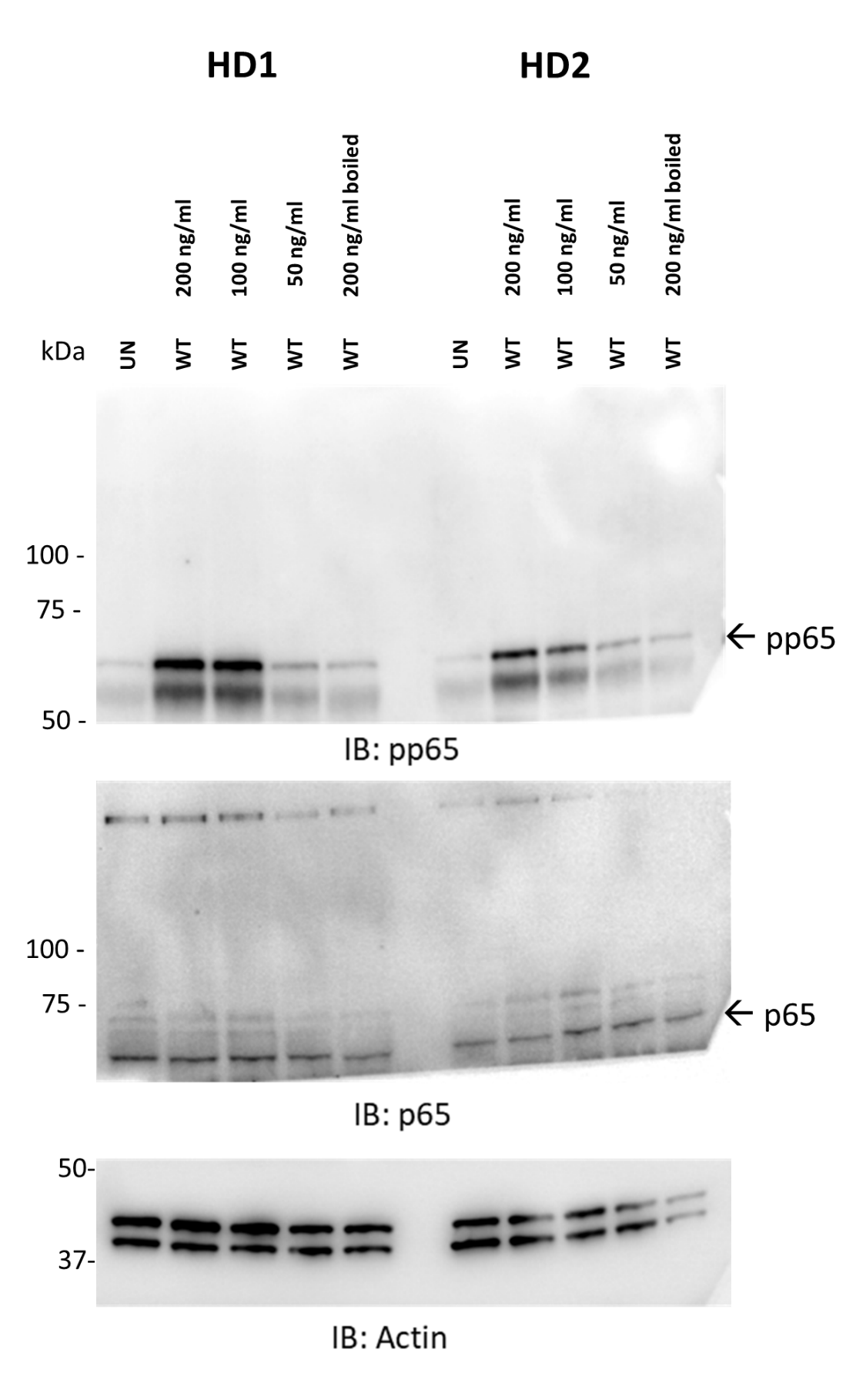


**Figure S6**. Wild-type (WT) NAMPT dose-dependent kinetics. Original western blot analysis of phospho(p)-p65 in two different healthy donor (HD) macrophage preparations upon treatment (20 minutes) with WT protein at concentration of 200-100-50 ng/ml, commonly used for triggering NF-KB pathway. We also included NAMPT boiled for 10 minutes, showing that it was unable to activate p-p65, thus ruling out endotoxin contaminations in the recombinant protein preparation. UN: untreated.


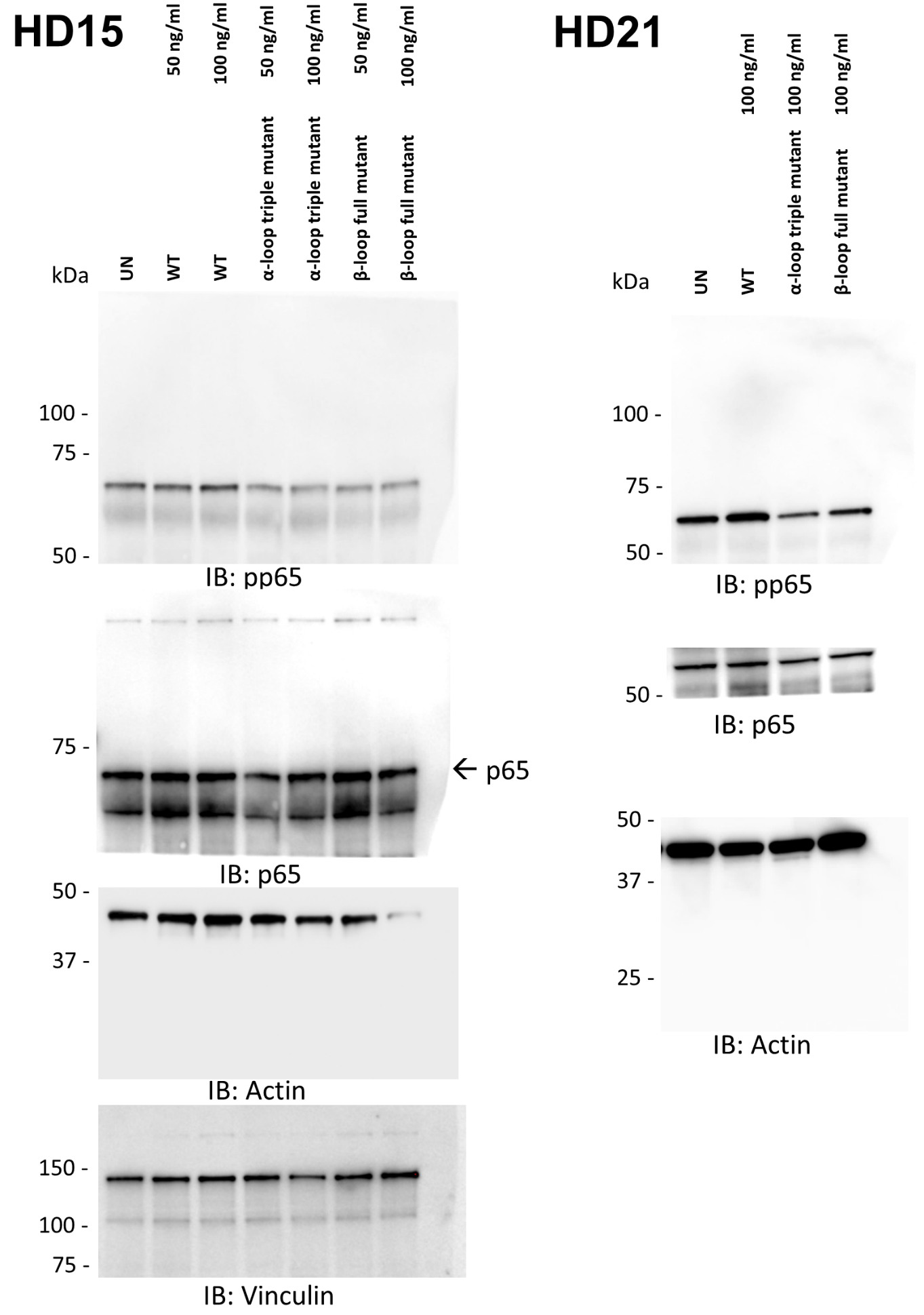


**Figure S7**. Original western blots of phospho(p)-p65 analyses in macrophages differentiated from two different healthy donors (HD). Figure 5 in the article is based on blot from HD21.


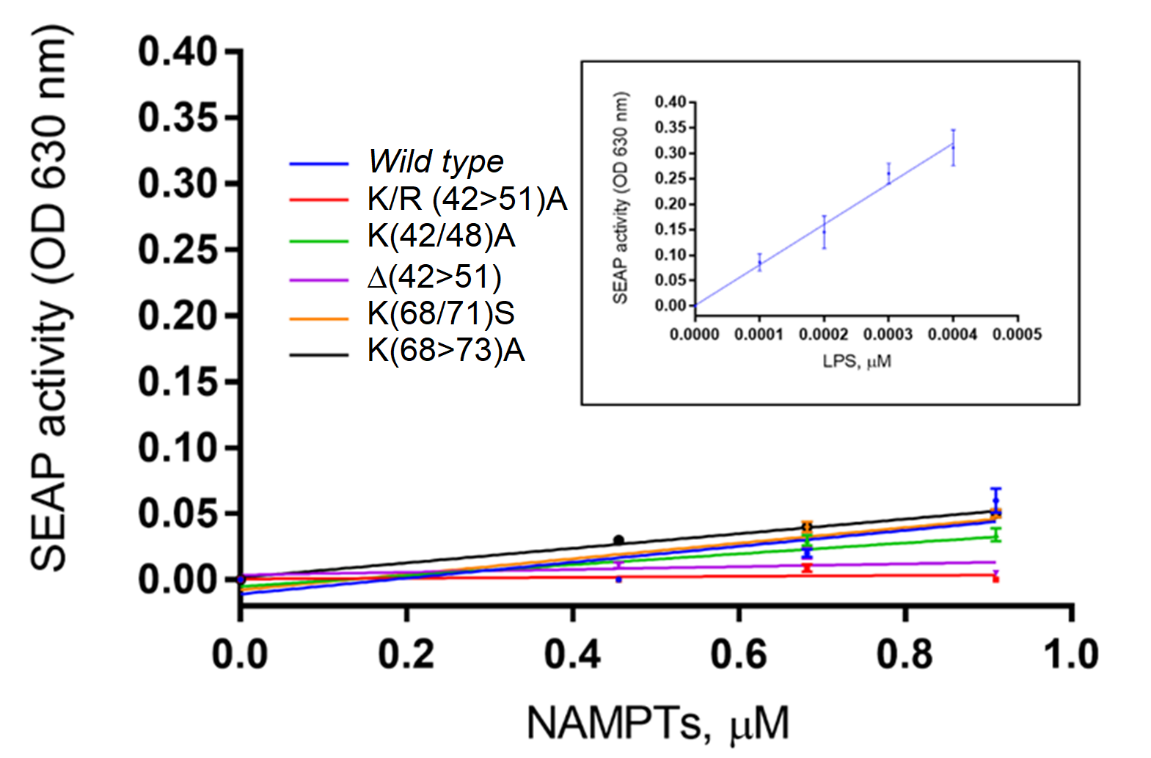


**Figure S8.** Effect of NAMPTs and LPS (inset) on SEAP secretion by HEK-Blue hTLR4 cells. Cells were exposed to the indicated concentrations of LPS or NAMPTs as described in Experimental Procedures.

**Table S1**. Main electrostatic interactions in the TLR4-NAMPT complex model. TLR4 residue position refers to the the human TLR4 sequence (O00206). The TLR4-NAMPT complex quaternary structure is constituted by two TLR4 subunits (sub A, sub B) and two NAMPT subunits (sub C, sub D).

| **TLR4** | | | **NAMPT** | | |
| --- | --- | --- | --- | --- | --- |
| **aa** | **#** | **sub** | **aa** | **#** | **sub** |
| Asp | 50 | A | Lys | 68 | C |
| Asp | 50 | A | Lys | 68 | D |
| Glu | 79 | A | Lys | 71 | D |
| Glu | 79 | A | Lys | 68 | D |
| Asp | 60 | A | Lys | 68 | D |
| Glu | 79 | A | Lys | 73 | D |
| Asp | 502 | A | Lys | 48 | D |
| Glu | 474 | A | Lys | 48 | D |
| Arg | 460 | B | Glu | 366 | D |

**Table S2**. Sequences of mutagenic primers used in this study.

| **mutation** | P | **sequence 5' 🡪 3'** |
| --- | --- | --- |
| **K(42/48)A** | fw | GAGTGCCGTGAAGCGAAGACAGAAAACTCCGCATTAAGGAAGGTGAAATATGAG |
|  | rw | CTCATATTTCACCTTCCtTAATGCGGAGTTTTCTGTCTTCGCTTCACGGCACTC |
| **K/R(42>51)A** | fw | CCTACTTTGAGTGCCGTGAAGCGGCGACAGAAAACTCCGCATTAGCGGCGGTGAAATATGAGGAAAC |
|  | rw | GTTTCCTCATATTTCACCGCCGCTAATGCGGAGTTTTCTGTCGCCGCTTCACGGCACTCAAAGTAGG |
| **Δ(42>51)** | fw | GCCGTGAAGTGAAATATGAGGAAACAGTATTTT |
|  | rw | ATTTCACTTCACGGCACTCAAAGTAGGA |
| **K(68/71)S** | fw | GGGCTGCAGTACATCCTTAATAGCTACTTAAGCGGTAAGGTAGTAACC |
|  | rw | GGTTACTACCTTACCGCTTAAGTAGCTATTAAGGATGTACTGCAGCCC |
| **K(68/71)A** | fw | GAGGAAACAGTATTTTATGGGTTGCAGTACATTCTTAATGCGTACTTAGCAGGTAAAGTAGTAACCAAAGAG |
|  | rw | CTCTTTGGTTACTACTTTACCTGCTAAGTACGCATTAAGAATGTACTGCAACCCATAAAATACTGTTTCCTC |
| **K(68>73)A** | fw | GAGGAAACAGTATTTTATGGGTTGCAGTACATTCTTAATGCGTACTTAGCAGGTGCAGTAGTAACCAAAGAGAAAATCCAG |
|  | rw | CTGGATTTTCTCTTTGGTTACTACTGCACCTGCTAAGTACGCATTAAGAATGTACTGCAACCCATAAAATACTGTTTCCTC |
| **K(68>77)A** | fw | GAGGAAACAGTATTTTATGGGTTGCAGTACATTCTTAATGCGTACTTAGCAGGTGCAGTAGTAACCGCAGAGAAAATCCAG |
|  | rw | CTGGATTTTCTCTGCGGTTACTACTGCACCTGCTAAGTACGCATTAAGAATGTACTGCAACCCATAAAATACTGTTTCCTC |
